# Differential patterns of blood oxygenation in the prefrontal cortex between patients with methamphetamine-induced psychosis and schizophrenia

**DOI:** 10.1038/srep12107

**Published:** 2015-07-16

**Authors:** Kazuhiko Yamamuro, Manabu Makinodan, Sohei Kimoto, Naoko Kishimoto, Tsubasa Morimoto, Michihiro Toritsuka, Kiwamu Matsuoka, Yoshihiro Takebayashi, Tomoyo Takata, Masato Takahashi, Yoshinori Tanimura, Yosuke Nishihata, Yasuhiro Matsuda, Toyosaku Ota, Hiroki Yoshino, Junzo Iida, Toshifumi Kishimoto

**Affiliations:** 1 Department of Psychiatry, Faculty of Medicine, Nara Medical University, Kashihara, Japan; 2 Faculty of Nursing, Nara Medical University, Kashihara, Japan

## Abstract

Despite some slight differences in symptomatology, differential diagnosis of methamphetamine-induced psychosis (MAP) versus schizophrenia can be challenging because both disorders present a large overlap in their clinical symptoms. However, a recent study has shown that near-infrared spectroscopy (NIRS) performed during a cognitive task can be a powerful tool to differentiate between these two disorders. Here, we evaluated verbal fluency task performance during NIRS in 15 patients diagnosed with MAP and 19 with schizophrenia matched for age and sex. We used prefrontal probes and a 24-channel NIRS machine to measure the relative concentrations of oxyhaemoglobin every 0.1 s during the task. For each patient, the neurocognitive function and clinical psychopathology were evaluated using the Positive and Negative Symptom Scale (PANSS), and the Brief Assessment of Cognition in Schizophrenia (BACS). Oxyhaemoglobin changes in the prefrontal cortex were significantly higher in the MAP group compared to those in the schizophrenia group, particularly in the right dorsolateral prefrontal cortex. In contrast, we found no significant difference in PANSS and BACS scores. Our findings suggest that NIRS measurement could be applied to differentiate patients with MAP from those with schizophrenia, even in cases where clinical symptoms are similar.

Methamphetamine (MA) intake causes psychotic symptoms such as paranoid delusions and hallucinations in some subjects that do not have pre-existing psychotic manifestations^1^,^2^. Methamphetamine-induced psychosis (MAP), which occurs in 10 to 60% of MA abusers^3^,^4^,^5^, is likely due to repeated administration or the use of high doses of MA^6^,^7^. MAP results in a lasting enhancement of dopamine release in the striatum and nucleus accumbens^8^, and therefore is usually well-treated with dopamine antagonists. On the other hand, schizophrenia is a psychiatric disorder that begins during adolescence, and is typically characterized by hallucinations and paranoid delusions. The dopamine hypothesis of schizophrenia is attributable to the principal descriptive model of antipsychotic drug action. Because of this, there are similarities between MAP and schizophrenia, which range from clinical symptomatology to pharmacotherapeutical characteristics^9^.

Schizophrenia patients present both positive and negative symptoms; however, even though MAP patients obviously suffer from positive symptoms similar to schizophrenia^10^, the existence of negative symptoms in these patients is controversial. Previous reports showed that the severity of psychotic symptoms including negative symptoms observed in MAP and schizophrenia are almost identical^11^,^12^, whereas the negative symptoms evaluated by the Scale for the Assessment of Negative Symptoms (SANS) differ^13^. MAP and schizophrenia are usually diagnosed based on their symptoms, and it can be difficult to distinguish schizophrenia from MAP or other drug use-associated disorders in case that the psychotic symptoms are clearly identical.

Patients with schizophrenia show a wide variety of neurocognitive deficits in verbal, working, and implicit memory and social cognition^14^,^15^, which are related to aberrant gamma-aminobutyric acid signalling in the prefrontal cortex^16^,^17^. In a similar manner, patients with MAP also show similar cognitive dysfunctions in working memory and other executive functions^18^,^19^; which makes it challenging to distinguish MAP and schizophrenia based on clinical symptoms^20^,^21^. Furthermore, both diagnostic groups also share other psychiatric symptoms, such as impaired inhibitory control and impulsivity^22^,^23^. Interestingly, clinical investigations have suggested that exposure to MA may cause a persistent schizophrenia-like psychosis, which stirs debate as to whether persistent MAP is distinct from schizophrenia^24^,^25^. In spite of this, few studies have investigated similarities and differences in brain dysfunction between MAP and schizophrenia. Recently, Okada *et al.* reported that using near-infrared spectroscopy (NIRS), only patients with MAP showed reduced activation in the frontopolar prefrontal cortex during the stop-signal inhibitory task^26^. It has been previously reported that patients with schizophrenia have significant cognitive dysfunctions^27^,^28^. Therefore, in this study, we monitored verbal fluency task (VFT) performance during NIRS and evaluated the potential application of this technique for the differential diagnosis of MAP and schizophrenia.

## Results

### Demographic and clinical data

The demographic characteristics of the study participants are presented in Table 1. There were no significant differences in age, sex, JART IQ, duration of illness, neuroleptics, number and duration of hospitalization between patients with MAP and schizophrenia. Moreover, there were no significant differences between both groups in PANSS or BACS subscales (Table 2).

### NIRS data from participants performing verbal fluency tasks

**Figure 1:**
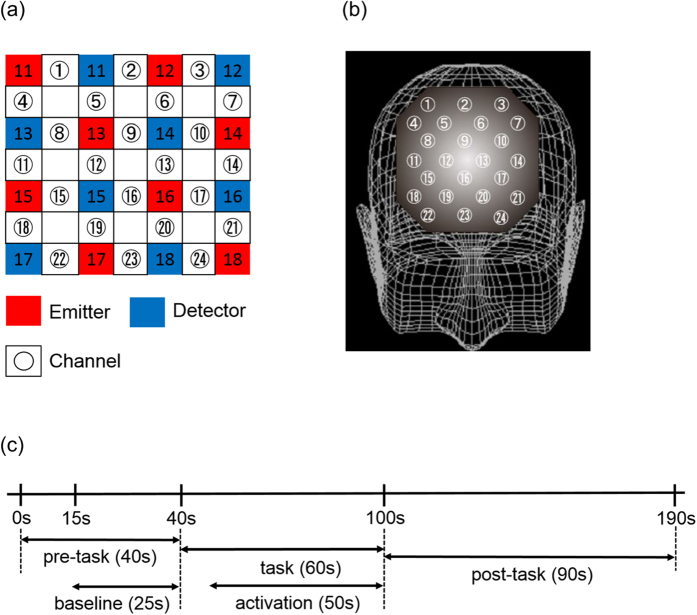
Location of the 24 channels in the near-infrared spectroscopy instrument: (**a**) Corresponding anatomical site of each channel. **(b)** Arrangement of emitters and detectors, as well as definition of each channel. **(c)** Protocols and procedures for data analysis in each task. Pre- and post-task periods for all verbal fluency tasks (VFT) performed were 30 s and 90 s, respectively. For data analysis, we defined baseline as the mean levels of oxyhaemoglobin changes during the last 25 s of the pre-task period. The mean levels of oxyhaemoglobin changes during the 60-s task period of VFT were defined as activation levels.

**Figure 2:**
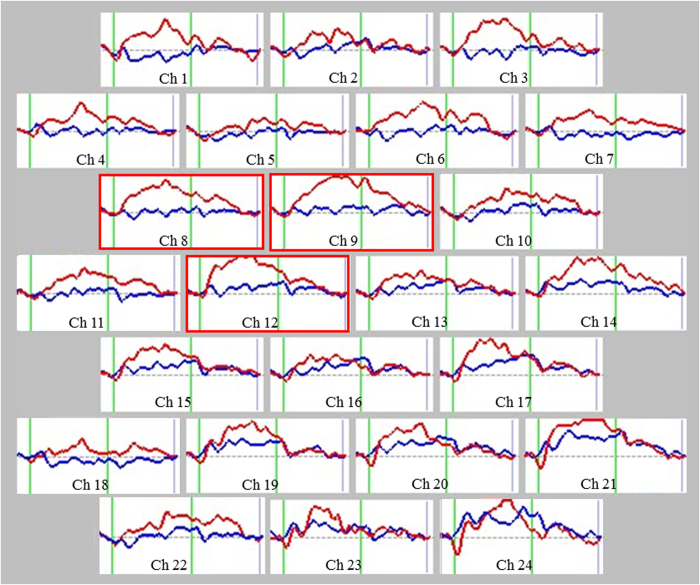
Grand average waveforms showing changes in oxyhaemoglobin during the VFT. Red lines represent the methamphetamine-induced psychosis group, while blue lines represent the schizophrenia group. The task was performed in the interval represented by green lines; the first green line indicates the beginning of the task and the second indicates the end. Abbreviations: Ch, channel.

We calculated the grand average waveforms of oxyhaemoglobin (oxy-Hb) concentration changes while patients of both groups performed verbal fluency tasks (VFT) (Fig. 1). The grand waveforms of oxy-Hb concentration change increased while participants in the MAP group were performing the task. In contrast, the grand waveforms of oxy-Hb concentration showed little change in the participants in the schizophrenia group. We found group differences in the mean oxy-Hb measurements between the pre-task and post-task periods in the 24 channels (Fig. 2) (FDR-corrected, all P = 0.002 ~ 0.008). In the MAP group, the mean change in oxy-Hb between the pre-task and post-task periods was significantly larger than in the schizophrenia group for channels 8, 9, and 12. Those channels were located near the right dorsolateral prefrontal cortex (DLPFC). Overall, while performing the VFT, the MAP group exhibited smaller oxy-Hb changes in the prefrontal cortex compared to the schizophrenia group, particularly in the right DLPFC.

### Correlation between NIRS measurements and demographic characteristics

Because MAP and schizophrenia patients varied considerably in terms of characteristics, we performed Spearman’s ρ correlation calculations for NIRS measurements (Channels 8, 9 and 12), age, JART IQ, duration of illness, neuroleptics, and PANSS subscales. There were no significant correlations between mean oxy-Hb changes and any other potential confounding factors, for any of the two groups.

**Figure 3:**
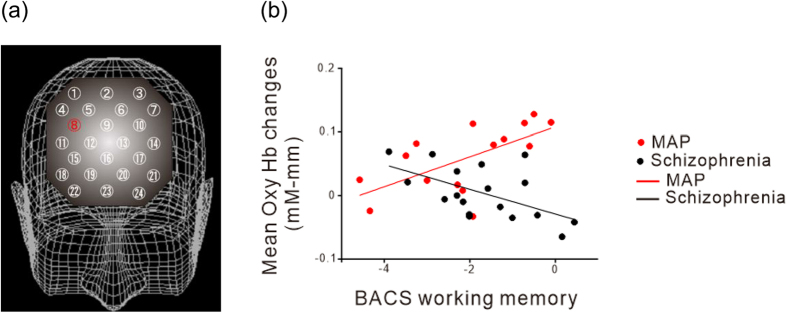
Comparison of the association between working memory and haemodynamic changes for the methamphetamine-induced psychosis and schizophrenia groups. (**a**) Three-dimensional topographical mapping of the channels showing the location of significant differences in correlation between the two groups (channel #8, labelled in red; uncorrected P = 0.001. (**b**) Scatterplot of a typical channel presenting significantly different correlations for the two groups [Ch 8 (MAP) r = 0.565, (schizophrenia) r = −0.550, Z = 3.170, P = 0.002]. Abbreviations: BACS, brief assessment of cognition in schizophrenia; MAP, methamphetamine-induced psychosis.

We then compared mean oxy-Hb changes and working memory BACS scores. In the MAP group, we found a significant positive correlation at channel 8 (ρ = 0.565, *p* = 0.035). On the other hand, in the schizophrenia group, we found a significant negative correlation at channel 8 (ρ = −0.550, *p* = 0.018) (Fig. 3).

## Discussion

To the best of our knowledge, this is the first study investigating prefrontal haemodynamic response during VFT between patients with MAP and schizophrenia using NIRS. We found that oxy-Hb changes in the prefrontal cortex (channels 8, 9 and 12) during VFT were significantly larger in patients with MAP than they were in patients with schizophrenia. Our results suggest that patients with MAP have significantly different blood oxygenation in the right DLPFC compared with that in patients with schizophrenia, and NIRS could be a useful measurement tool to distinguish the two disorders, which is consistent with the findings of a previous report^26^.

A number of researchers have used NIRS for the assessment of schizophrenia symptoms, although only one study has been published. Okada *et al.* reported that using NIRS, patients with MAP exhibited significantly smaller changes in oxy-Hb at the frontopolar prefrontal cortex during stop-signal tasks, and higher impulsivity compared with patients with schizophrenia^26^. These findings are reasonable because the frontopolar cortex and its junction with the temporal lobe are considered the neuronal basis of impulsivity and moral judgment^29^,^30^,^31^. On the other hand, NIRS studies revealed that patients with schizophrenia show a reduction in prefrontal activation during the performance of VFT^27^,^28^. During VFT, which is a task of executive function, subjects are asked to generate as many words as possible for a given letter or category, during a limited time period. This requires a wide range of cognitive abilities, including evocation of appropriate words, suppression of incorrect words, remembering which words have already been used, and attention to the task^32^,^33^. Patients with schizophrenia show poor performance in this complex task^34^, as well as low oxygenation of the prefrontal cortex. Interestingly, it had been never investigated how the prefrontal cortex responds to VFT in patients with MAP. Therefore, we examined haemodynamic changes during the performance of VFT in patients with MAP and schizophrenia. In the DLPFC, patients with schizophrenia exhibited significantly smaller changes in oxy-Hb compared to MAP patients. Since DLPFC is a region responsible for working memory, and impaired working memory is a core symptom of schizophrenia^35^,^36^,^37^, hypo-oxygenation in the DLPFC in these patients can be reasonably expected. In spite of differential oxygenation of the DLPFC, scores of working memory in BACS domains were low in patients with both MAP and schizophrenia in this study, which is consistent with a previous study^38^.

A previous report showed that patients with MAP and those with schizophrenia presented an opposite correlation between impulsivity and haemodynamic changes in the bilateral premotor cortex^26^. Similar to these findings, our results showed that patients with MAP exhibited an opposite correlation between working memory and haemodynamic changes in channel 8 compared to patients with schizophrenia; there was a positive correlation in patients with MAP, while a negative correlation was observed in patients with schizophrenia. The interesting point in our study is that scores of working memory in BACS were identical for the two disorders, while scores of excitement (impulsivity) in PANSS were higher in MAP than in schizophrenia in a previous study^24^, which suggests that our NIRS analysis during VFT was able to distinguish between the two groups, even in cases in which clinical symptoms were completely identical.

There are several potential limitations in our study. First, the spatial resolution for the detection of haemodynamic responses from the scalp surface using NIRS is lower than that of more deeply penetrating functional imaging such as functional MRI. However, this limitation could be within an acceptable range because the differences in haemodynamic response in individuals with MAP and schizophrenia could be clearly observed using NIRS, although it would be interesting to investigate activity differences of the whole brain using functional MRI. Second, our sample size was relatively small, and future studies should include a larger sample size to examine if such changes measured by NIRS are dose-dependent in patients with MAP. Third, all participants were receiving antipsychotic medications during the tests, although there was no significant difference in drug dose between the two groups.

## Conclusion

To the best of our knowledge, this is the first NIRS study to examine prefrontal haemodynamic responses during the performance of VFT in participants with MAP and schizophrenia. We found that changes in oxy-Hb concentration in the prefrontal cortex were significantly higher in patients with MAP compared to those with schizophrenia. Our study indicates that patients with MAP might have less prefrontal dysfunction than patients with schizophrenia. Since the multi-channel NIRS system enables non-invasive functional mapping of the cerebral cortex, and the measurement time is much shorter (about 5 min) than that in other functional brain imaging methodologies, this could be a powerful tool for the differential diagnosis of MAP and schizophrenia in cases that present similar clinical symptoms.

## Methods

### Participants

Fifteen MAP patients (9 males: mean ± SD age 42.6 ± 11.6 years; and 6 females: mean ± SD age 35.8 ± 9.3 years), and 19 schizophrenia patients (9 males: mean ± SD age 37.3 ± 8.0 years; and 10 females: mean ± SD age 40.7 ± 6.0 years) were recruited from the in- and outpatient clinics of the Department of Psychiatry at Nara Medical University, Japan (Table 1). For each patient, diagnosis was determined according to DSM-5 and ICD-10 research criteria for schizophrenia or methamphetamine psychosis. At least two experienced psychiatrists separately examined the patients, and diagnostic consensus was reached. All participants were of Japanese descent and right-handed, as indicated by the Edinburgh Handedness Inventory^39^. Their premorbid IQs were assessed using the Japanese version of the National Adult Reading Test (JART)^40^. Exclusion criteria included neurological disorders, traumatic brain injury with known cognitive consequence or loss of consciousness for more than 5 min, history of electroconvulsive therapy, history of pervasive developmental disorder, a serious medical condition, or low intelligence. Seventeen out of the 19 patients with schizophrenia received atypical neuroleptics, while the other 2 patients received both typical and atypical neuroleptics. On the other hand, all patients with MAP received atypical neuroleptics. Mean chlorpromazine equivalent dosages for antipsychotics did not differ between subject groups.

The study protocols were approved by the appropriate Ethics Committees at Nara Medical University (No. 325-2) and was carried out in accordance with the Declaration of Helsinki. All study participants or their legal guardians provided informed consent for their participation prior to the start of the study.

### Neurocognitive evaluation

The Brief Assessment of Cognition in Schizophrenia (BACS) scale was used to evaluate the neurocognitive functions of all patients^41^. BACS is a newly developed instrument that evaluates the elements of cognition that are most commonly impaired and strongly connected with real world functioning aspects in patients with schizophrenia^42^. The BACS evaluation takes about 30 min, yields a high completion rate in patients, and has high test-retest reliability. BACS evaluation included such tests as the List Learning Test, Digit Sequencing Task, Token Motor Task, Category Instances Test, Controlled Oral World Association Test, Symbol Coding, and Tower of London Test, which measure verbal memory, working memory, motor speed, verbal fluency, attention, processing speed, and executive function, respectively. The primary measure of each BACS domain was standardized creating z-scores, and the composite score is the Z-score of that sum^43^.

### Psychopathological evaluation

The Japanese version of the Positive and Negative Symptom Scale (PANSS)^44^ was used to evaluate symptoms in the participants with schizophrenia and MAP. A seven point Likert-scale, in which higher scores indicate greater severity, was used to rate all of the items. Subscale scores were calculated using small sets of variables based on the three domains of PANSS: positive, negative, and general psychopathological symptoms^45^.

### Cognitive activation task

Patients were administered the 160→190 block VFT (letter version). In the present study, we chose VFT because previous studies have demonstrated deficits in brain cortical activity over bilateral frontotemporal regions during VFT in patients with schizophrenia, using NIRS measurement. The 190-s block VFT contains three different time periods: a 40-s pre-task, a 60-s task, a 90-s post-task periods. In the 25→40 pre-task and 90-s post-task periods, patients were instructed to say Japanese vowels (/a/, /i/, /u/, /e/, and /o/) repeatedly to control for, and remove task-related motion artefacts during the task periods. During the 60-s task period, patients were instructed to say as many words as possible that started with a phonological syllable presented as an audible instruction by a computer. The task period comprised three continuous 20-s subperiods (/a/. /ki/, and /ha/). Transitions between the 20-s subperiods were continuous, which encouraged patients to produce continuous vocalizations (Fig. 1(c)). We recorded the total number of correct words generated during the task as an index of VFT performance.

### NIRS measurements

Oxy-Hb increases and deoxy-Hb decreases, as measured using NIRS, have been shown to reflect cortical activation. In animal studies, oxy-Hb has been shown to be the most sensitive indicator of regional cerebral blood flow, because the direction of change in deoxy-Hb is determined by the degree of change in venous blood oxygenation and volume^46^. Therefore, we decided to focus on changes in oxy-Hb. In this study, oxy-Hb was measured with a 24-channel NIRS machine (Hitachi ETG-100, Hitachi Medical Corporation, Tokyo, Japan), determining the absorption of two wavelengths of near infrared light (760 and 840 nm). Oxy-Hb was calculated as previously described^47^. The inter-probe intervals of the machine were 3.0 cm, and it was determined that the machine measured points 2-3 cm beneath the scalp, which is the depth of the surface of the cerebral cortices^48^. Participants maintained a natural sitting position during NIRS measurements. The NIRS probes were placed on the participant’s prefrontal regions, and were arranged to measure relative oxy-Hb concentration changes at 24 measurement points in an 8 × 8 cm area (Fig. 1(a)). The lowest probes were positioned along the Fp1-Fp2 line, according to the international 10–20 system used in electroencephalography. The probe positions and measurement points on the cerebral cortex were confirmed by overlaying the probe positions on a three-dimensionally reconstructed magnetic resonance imaging (MRI) scan of the cerebral cortex of a representative participant from the control group (Fig. 1(b)). The absorption of near-infrared light was measured with a time resolution of 0.1 s, and data were analysed using the “integral mode”. The pre-task baseline was determined as the mean during the 10 s immediately preceding the task performance, and the post-task baseline was determined as the mean during the 25 s immediately following completion of the task. Linear fitting was performed on the data between the two baselines. Moving average methods (moving average window, 5 s) were used to exclude short-term motion artefacts in the analysed data. We tried to minimize motion artefacts by closely monitoring subjects, and by instructing them to avoid artefact-evoking body movements, such as neck movements, strong biting, and blinking. Particular attention was paid to blinking because it was identified as the most influential source of motion artefacts in a preliminary artefact-evoking study. Examiners who were blind to the diagnosis of the participants evaluated the NIRS results.

### Statistical Analyses

We used Student’s *t*-tests to compare oxy-Hb changes between the two groups by calculating the grand average waveforms every 0.1 s in each channel. This analysis enabled a more detailed comparison of oxy-Hb changes along the time course of the task. Since we performed 24 paired *t* tests, we performed a correction for multiple comparisons using the false discovery rate (FDR) (two-tailed; we set the value of *q* specifying the maximum FDR to 0.05, so that on average, there would be no more than 5% false positives^49^). We used PASW Statistics 18.0 J for Windows (SPSS, Tokyo, Japan) for statistical analysis.

## Additional Information

**How to cite this article**: Yamamuro, K. *et al.* Differential patterns of blood oxygenation in the prefrontal cortex between patients with methamphetamine-induced psychosis and schizophrenia. *Sci. Rep.* **5**, 12107; doi: 10.1038/srep12107 (2015).

## Figures and Tables

**Table 1:** Participant characteristics.

Variable	Patients with MAP (n = 15)		Patients with schizophrenia (n = 19)		P value
	Mean	SD	Mean	SD	
Age	39.87	11.20	39.11	7.01	0.81
Male/female	9/6		9/10		
JART IQ	95.88	12.02	100.30	13.79	0.25
Age at first use of methamphetamine	21.81	5.54	NA	NA	NA
Age of onset of psychotic symptoms	33.19	12.70	NA	NA	NA
Duration of illness	8.42	5.09	13.80	10.69	0.08
Neuroleptics (chlorpromazine equivalent)	505.95	456.28	572.50	382.37	0.35
Biperiden	0.57	0.94	1.55	2.94	0.24
Number of hospitalizations	2.50	3.54	1.47	1.26	0.24
Duration of hospitalizations (months)	8.41	5.09	12.17	28.73	0.98

MAP, methamphetamine induced psychosis; JART, Japanese Adult Rating Test; SD, standard deviation; NA, not applicable.

**Table 2:** Participant symptom scores.

Variable	Patients with MAP (n = 15)		Patients with schizophrenia (n = 19)		P value
	Mean	SD	Mean	SD	
**PANSS**					
Positive	15.27	4.70	14.00	3.97	0.39
Negative	16.40	7.15	19.63	6.10	0.16
General psychopathology	37.23	9.92	41.11	8.90	0.22
**BACS**					
Verbal memory	−2.17	1.38	−1.86	0.97	0.34
Working memory	−1.67	1.06	−1.23	1.11	0.24
Motor speed	−3.20	1.54	−2.65	1.22	0.21
Verbal fluency	−1.35	1.16	−1.03	0.66	0.23
Attention and processing speed	−2.27	1.41	−1.73	0.86	0.19
Executive function	−1.83	2.39	−0.86	2.20	0.28
Composite score	−2.10	1.24	−1.56	0.78	0.16

MAP, methamphetamine-induced psychosis; PANSS, Positive and Negative Symptoms Scale; BACS, Brief Assessment of Cognition in Schizophrenia.
